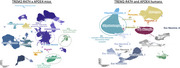# Single‐cell transcriptomics analysis identifies oligodendrocyte‐microglia crosstalk modulated by TREM2‐R47H and exacerbated by APOE4 in Alzheimer's disease

**DOI:** 10.1002/alz70855_106895

**Published:** 2025-12-24

**Authors:** Diana J Zajac, Gillian K Carling, Li Fan, Jielin Xu, Li Gan, Feixiong Cheng

**Affiliations:** ^1^ Cleveland Clinic, Cleveland, OH, USA; ^2^ Weill Cornell Medicine, New York, NY, USA; ^3^ Helen and Robert Appel Alzheimer's Disease Research Institute, Brain and Mind Research Institute, Weill Cornell Medicine, New York, NY, USA; ^4^ Cleveland Clinic Genome Center, Cleveland, OH, USA

## Abstract

**Background:**

Alzheimer's Disease (AD) risk variants *APOE4* and *TREM2‐R47H* are known to impact glial cell functions and transcriptional profiles. *TREM2*‐mediated oligodendrocyte‐microglial crosstalk has not been revealed in previous research studies. Here, we present novel findings suggesting a *TREM2*‐dependent mediation of oligodendrocyte transcriptional profiles.

**Methods:**

We investigated cell‐type specific transcriptional changes associated with humanized *APOE4* and *TREM2‐R47H* genotypes in P301S+ mice versus background controls via single‐nuclei RNA‐sequencing of the frontal cortex. We investigated cell type subcluster abundance in association with genotype, sex and tau‐positivity. We classified subclusters through differentially expressed gene network modules and gene set enrichment analysis. We compared our findings in mice to a human cohort. The mouse cohort consisted of 52 humanized *APOE*x*TREM2* mice, with six‐to‐eight mice per genotype‐tau group, evenly split on sex. The human cohort consisted of 55 AD and control humans: *APOE4‐carrier TREM2‐R47H* (*E4‐R47H*) (*n* = 18), *nonE4‐R47H (nonE4‐R47H)* (*n* = 6), *APOE4‐carrier TREM2‐common variant* (*E4‐CV)* (*n* = 16), *nonE4‐CV* (*n* = 6).

**Results:**

We found that *APOE‐TREM2* status had sex‐ and tau‐independent *TREM2‐R47H*‐specific effects, and *APOE4‐R47H* synergistic effects on cell abundance in oligodendrocytes, oligodendrocyte progenitor cells (OPCs), and a subgroup of inhibitory neurons. Specifically, we identified OPC and oligodendrocyte subclusters that strongly associate with *TREM2‐R47H* in a tau‐independent manner, further exacerbated by *APOE* genotype. Of note, the human cohort also showed a strong synergistic effect of *APOE4* and *TREM2‐R47H* on an oligodendrocyte subcluster that may be related to the *TREM2‐R47H*‐dependent oligodendrocyte subclusters identified in the mice. This finding of *TREM2*‐specific effects on oligodendrocyte transcriptional states in both mice and humans suggests possible *TREM2*‐mediated microglia‐oligodendrocyte crosstalk, or the presence of *TREM2*‐oligodendrocyte cell‐ligand interactions that merits further investigation.

**Conclusion:**

In summary, this study suggests possible synergistic effects of *APOE4*‐carrier and *TREM2‐R47H*‐carrier status on OPC, oligodendrocyte, and inhibitory neuron abundance and transcriptional profiles. Specifically, OPCs and oligodendrocytes show a strong association with *TREM2‐R47H* that is exacerbated by *APOE* genotype, a finding that is reflected in the human cohort. Hence, further classification of differences between joint *APOE‐TREM2* genotypes will improve our understanding of glial cell alterations in AD, and could lead to novel cell type‐specific therapeutic interventions for AD and other AD‐related dementia if broadly applied.